# Post-translational modifications in CD8^+^ T cell exhaustion: mechanisms, networks, and epigenetic locking

**DOI:** 10.3389/fimmu.2026.1913598

**Published:** 2026-08-12

**Authors:** Ting Yu, Mengjuan Xia, Yuxia Wu, Qiongshi Wu, Jing Xie, Haocheng Gao, Shengfeng Hu

**Affiliations:** 1 Department of Pharmacy, Hainan General Hospital/Hainan Medical University Hainan Hospital, Haikou, China; 2 Medical Laboratory Center, Hainan General Hospital/Hainan Medical University Hainan Hospital, HaiKou, China; 3 Department of Gynecology, Hainan General Hospital/Hainan Medical University Hainan Hospital, HaiKou, China; 4 Guangdong Provincial Key Laboratory of Allergy & Clinical Immunology, The Second Affiliated Hospital, The Second School of Clinical Medicine, The State Key Laboratory of Respiratory Disease, School of Basic Medical Sciences, Guangzhou Medical University, Guangzhou, China

**Keywords:** cancer immunotherapy, CD8^+^ T cells, epigenetic locking, post-translational modifications, t cell exhaustion

## Abstract

CD8^+^ T cells are core effector cells of adaptive immunity. However, in chronic infection and tumor microenvironments, they often lose their effector functions. Under persistent antigenic and metabolic stress, CD8^+^ T cells may progressively acquire a stable dysfunctional state termed T cell exhaustion. Post-translational modifications (PTMs) constitute a rapid and dynamic regulatory system that targets four core functional modules: membrane receptor signaling, transcription factors, metabolic enzymes, and epigenetic regulators. These modules are interconnected through a multi-layered PTM network. Within this network, PTMs orchestrate a stepwise cascade that proceeds from signal initiation through metabolic reprogramming to transcriptional and epigenetic remodeling. Ultimately, this cascade consolidates the exhaustion program via stable epigenetic memory. Accordingly, we propose a “PTM-driven signal–metabolism–epigenetic locking model” as the central framework of this review. Focusing on five key PTMs, namely phosphorylation, ubiquitination, glycosylation, acetylation, and lactylation, we elucidate how these modifications cooperatively regulate CD8^+^ T cell exhaustion. Critically, the PTM network drives progressive locking of the exhausted state through three epigenetic layers: DNA methylation, chromatin remodeling, and histone modifications. These layers collectively contribute to the progressive stabilization of the exhausted state over time. This framework integrates transient environmental signals, metabolic fluctuations, and transcriptional changes into permanent cell fate decisions. Building upon this mechanistic understanding, the review further explores novel strategies targeting the PTM network to reverse exhaustion and enhance immunotherapeutic efficacy. This review provides a clear and comprehensive framework for understanding how PTMs govern CD8^+^ T cell exhaustion and lays a solid foundation for the development of next-generation immunotherapies.

## Graphical Abstract

**Figure:**
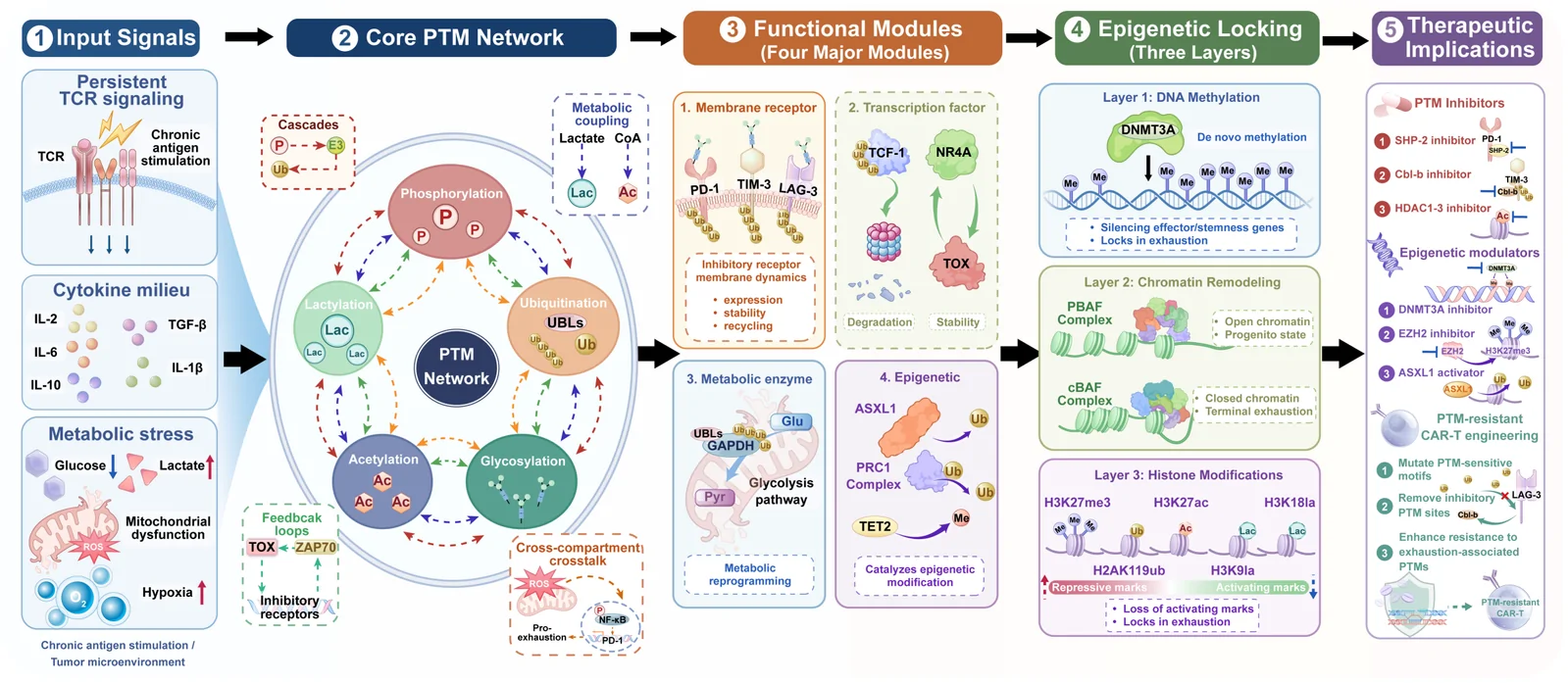
The PTM-driven signal–metabolism–epigenetic locking model of CD8^+^ T cell exhaustion.

## Introduction

CD8^+^ T cells serve as the core executors of adaptive immune responses and are essential for controlling acute infections and eliminating tumor cells (1). However, in chronic infections or tumor microenvironments (TME), persistent antigen exposure and metabolic constraints can induce CD8^+^ T cells to enter a special dysfunctional state called “T cell exhaustion” (2). Exhausted T cells (Tex) are characterized by sustained upregulation of inhibitory receptors (IRs), metabolic reprogramming, and profound transcriptional and epigenetic alterations (3). Clinically, T cell exhaustion acts as a major obstacle to cancer immunotherapy, seriously limiting the long-term efficacy of immune checkpoint blockade (ICB) and adoptive T cell therapy (4). Therefore, elucidating the core regulatory network of T cell exhaustion represents a fundamental scientific challenge for overcoming the bottlenecks of immunotherapy and improving clinical outcomes.

Post-translational modifications (PTMs) represent the core rapid-response mechanism that regulates T cell signal transduction and functional remodeling, and they are crucial for maintaining normal immune responses and pathological dysfunction (5, 6). In recent years, researchers have paid widespread attention to the role of PTMs in determining CD8^+^ T cell fate and driving the occurrence and development of exhaustion (7). For example, phosphorylation directly regulates the signal activity of IRs such as PD-1 (8); ubiquitination and ubiquitin-like modifications (UBLs) such as UFMylation determine the stability and membrane localization of receptor proteins (9); metabolic stress in the tumor microenvironment can also convert transient stimuli into stable epigenetic memory through modifications such as histone acetylation and lactylation, ultimately locking the exhaustion program (10). Despite these advances, most existing studies have focused on individual PTMs in isolation, leaving a gap in our understanding of how different PTMs coordinate with each other across functional modules and over time.

To address this gap, this review focuses on five core PTMs, namely phosphorylation, ubiquitination, acetylation, glycosylation, and lactylation, and follows the main line of functional module regulation, integrated network interaction, and epigenetic memory locking. We clarify how these PTMs regulate four core functional modules, including membrane receptor signals, transcription factors, metabolic enzymes, and epigenetic regulators, to form a synergistic network that progressively consolidates the exhausted state through epigenetic means. Based on these insights, we propose a “PTM-driven signal–metabolism–epigenetic locking model” that integrates the regulatory networks of distinct PTMs and provides a new theoretical framework and potential therapeutic targets for cancer immunotherapy.

## Overview of PTMS in T cell exhaustion

PTMs are the core mechanism for rapid functional regulation after protein synthesis, enabling rapid responses to extracellular signals and metabolic states without changing the level of gene transcription (11). They are regulated by the highly conserved “Writer-Eraser-Reader” enzyme system. This system achieves precise and reversible site-specific control (12). Writers add chemical groups, Erasers remove them, and Readers interpret these marks to modulate protein activity, localization, interactions, and gene expression (12–14). This coordination makes PTMs dynamic molecular switches rather than static markers.

A distinctive feature of PTMs is that many are directly driven by metabolites. Metabolite-driven PTMs (such as acetylation, lactylation, palmitoylation, and NEDDylation) act as the core hub connecting cellular metabolism and immune signals (15). During CD8^+^ T cell exhaustion, metabolic intermediate abundance determines PTM donor availability, regulating PTM intensity and specificity and forming a feedback loop linking metabolism, PTM activity, and exhaustion (16).

Thus, PTMs act as a molecular bridge linking transient metabolic and signal input to persistent functional output. These effects converge on four core nodes: membrane receptor signals, transcription factor activity, metabolic enzyme behavior, and epigenetic regulation (17, 18). Metabolic intermediates such as acetyl-CoA, UDP-GlcNAc, and lactate directly serve as donor substrates for PTMs (15, 19), highlighting PTMs as a key integrative node within the signal–metabolism–epigenetic axis.

## PTM regulation of four core functional modules

**Figure 1:**
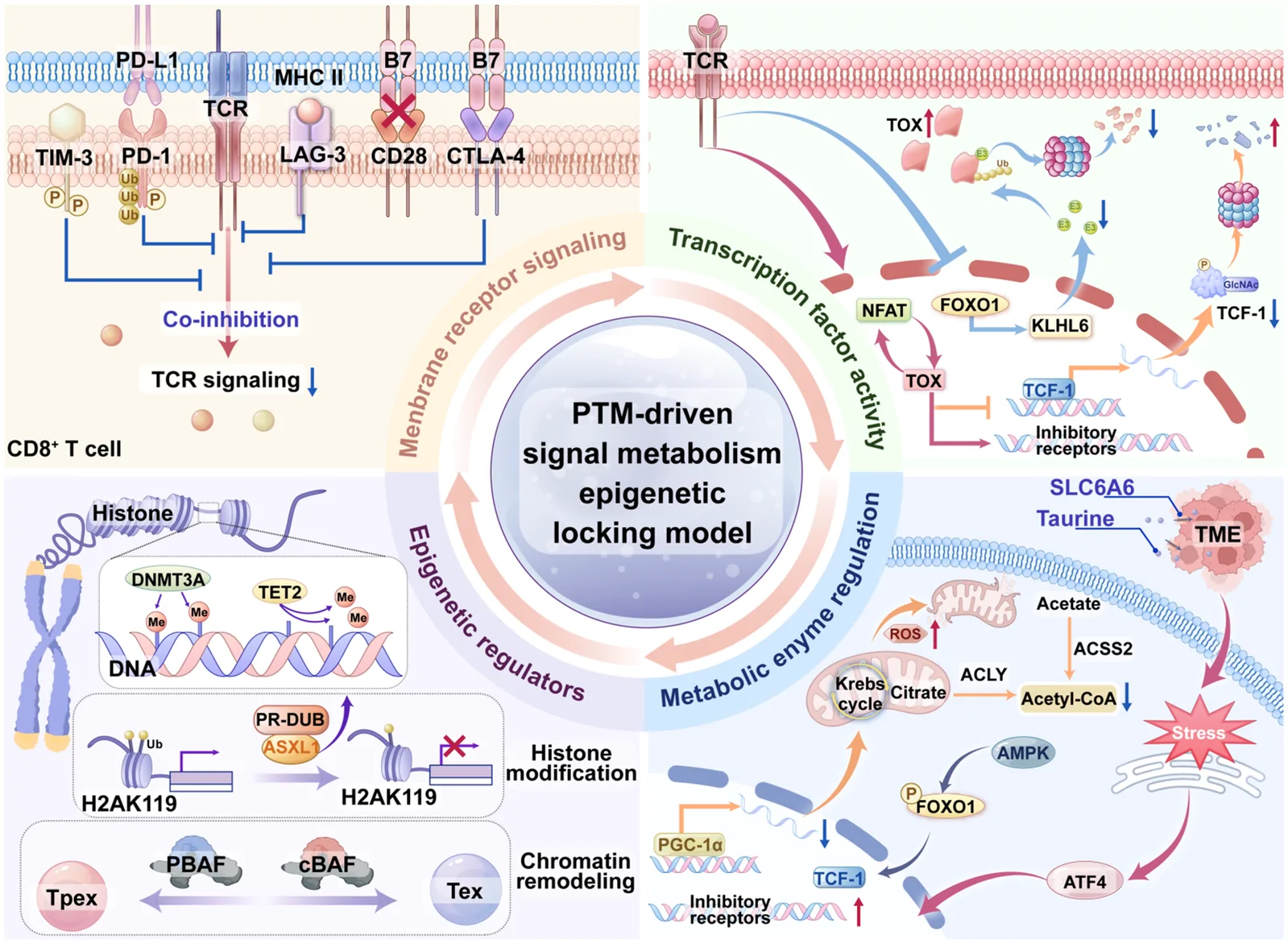
The PTM-driven signal–metabolism–epigenetic locking model of CD8^+^ T cell exhaustion: four core functional modules. The schematic illustrates the PTM-driven signal–metabolism–epigenetic locking model, which integrates four core functional modules to orchestrate exhaustion progression. At the center, the model title is displayed. The four modules are color-coded: membrane receptor signaling in orange, transcription factor activity in green, metabolic enzyme regulation in blue, and epigenetic modifier behavior in purple. Solid arrows indicate the directional cascade: signal input–transcriptional regulation–metabolic reprogramming– epigenetic locking. Membrane receptor signaling converts sustained TCR stimulation into phosphorylation cascades, activating transcription factors (NFAT, TOX) to drive exhaustion-associated gene expression. PTMs on metabolic enzymes shift the cell toward an energy-deficient state, and epigenetic modifiers establish repressive chromatin marks (H3K27me3, H2AK119ub) that lock exhaustion and silence effector genes. Thus, PTMs across four modules collectively convert transient environmental signals (persistent TCR stimulation, cytokine milieu, metabolic stress) into permanent cell fate decisions.

CD8^+^ T cell exhaustion is a multi-dimensional dysfunctional process. PTMs orchestrate protein activity and signal transduction by targeting four core functional modules: membrane receptor signaling, transcription factors, metabolic enzymes, and epigenetic regulators. We systematically dissect the regulatory mechanisms of PTMs in CD8^+^ T cell exhaustion (**Figure 1**).

### Regulation of membrane receptors and proximal signals

Proximal membrane signal transduction directly regulates the initiation and maintenance of T cell exhaustion by reshaping the activation threshold of CD8^+^ T cells (20). In chronic infections and TME, persistent T cell receptor (TCR) signals act as the core initiating signal driving exhaustion (21, 22). Notably, recent studies have demonstrated that precursors of exhausted T cells can be identified early during acute infections, but these cells only acquire a complete exhaustion program upon sustained antigen stimulation (22, 23).

Persistent TCR signals significantly upregulate IRs such as PD-1, CTLA-4, LAG-3, and TIM-3. These receptors form an inhibitory network that increases the T cell activation threshold and establishes immune tolerance (24). After binding to ligands such as PD-L1, it strongly blocks TCR and CD28 signaling, inhibiting cytokine synthesis and cytotoxic functions (25, 26). CTLA-4 reinforces exhaustion by competitively antagonizing CD28 (27, 28). LAG-3 binding to MHC II interferes with calcium influx and cytokine secretion (29). TIM-3 undergoes conformational changes upon ligand binding, transforming into an inhibitory receptor that blocks effector function (30).

In this process, PTMs precisely control the protein stability, membrane localization, ligand affinity, and downstream signal intensity of IRs. For example, the protein level and signal output intensity of PD-1 are coordinately regulated by phosphorylation and ubiquitination, which is a key link in maintaining the stability of exhaustion signals (31); S-palmitoylation of CD80 determines its membrane localization and stability, thereby indirectly regulating the inhibitory efficacy of CTLA-4 (32); intracellular phosphorylation of TIM-3 directly determines its functional switch from “co-stimulation” to “co-inhibition” (30).

Overall, PTMs precisely control the intensity, duration, and sensitivity of membrane receptor signals. They thus serve as key molecular switches that determine whether CD8^+^ T cells undergo exhaustion or retain effector function under persistent stimulation.

### Transcription factors and intracellular signal transduction

A hierarchical transcription factor network governs the fate, differentiation, and function of exhausted CD8^+^ T cells (33, 34). Under persistent chronic antigen stimulation, this system deviates from the classic effector or memory T cell pathways and initiates an exclusive exhaustion transcriptional program.

Among the transcription factors involved, TOX is a recognized core master regulator. TOX drives three key processes: establishing the exhausted phenotype, maintaining functional states, and solidifying epigenetic programs (35, 36). Importantly, TOX forms a positive reinforcement loop with the NR4A family and NFAT, activating immune receptors including PD-1 and continuously strengthening the exhaustion program (37). At the same time, TOX can significantly antagonize the memory stemness-related transcriptional program dominated by TCF-1. By inhibiting stemness genes and activating terminal exhaustion genes, it promotes progenitor-like exhausted T cells (Tpex) to gradually lose stemness plasticity and directionally differentiate into terminally Tex with severely impaired functions (38). The balance between these two opposing axes determines Tex differentiation and function.

PTMs regulate key exhaustion-related transcription factors (TOX, TCF-1, STAT5A) via phosphorylation, ubiquitination, acetylation, and glycosylation, affecting their stability, localization, DNA binding, and activity. The ubiquitin-proteasome system controls TOX stability: KLHL6 ubiquitinates TOX for degradation, while persistent TCR signaling inhibits FOXO1-KLHL6, promoting TOX accumulation and exhaustion (39). TCF-1 is regulated by phosphorylation, acetylation, and ubiquitination, with chronic stimulation promoting its degradation and terminal exhaustion (38). STAT5A antagonizes exhaustion through tyrosine/serine phosphorylation and O-GlcNAc glycosylation, which control its nuclear translocation and activity (40, 41).

Thus, PTMs dynamically fine-tune the transcription factor network in a fast, reversible, and site-specific manner, without altering gene transcription levels. By integrating persistent antigen stimulation, cytokine signals, and metabolic stress into a unified transcriptional output, PTMs ultimately determine the differentiation trajectory, exhaustion degree, and lineage characteristics of CD8^+^ T cells under chronic stimulation.

### Metabolic enzymes and nutrient sensing

CD8^+^ T cells shift from an energy-efficient state to energy deficiency during exhaustion (42). Under persistent antigen stimulation and TME pressure, they develop metabolic disorders including impaired nutrient uptake, mitochondrial damage, respiratory dysfunction, and ROS accumulation, leading to irreversible effector decline (42, 43). PGC-1α downregulation is central to mitochondrial collapse, blocking oxidative phosphorylation and worsening metabolic imbalance (44, 45). Nutrient competition in the TME further impairs T cell metabolism, as exemplified by SLC6A6-mediated taurine depletion, which triggers ER stress and ATF4-driven ISR, upregulating IRs and accelerating terminal exhaustion (46).

PTMs serve as the core bridge linking metabolism to immune function. They dynamically regulate mTOR, AMPK (47), and metabolic enzymes in glycolysis, the TCA cycle, and mitochondrial homeostasis (48). The abundance of metabolites directly affects the availability of PTM donors such as acetyl-CoA and UDP-GlcNAc. This allows metabolic signals to quickly convert into protein modification changes, thereby regulating downstream signals and transcriptional programs (19). ACLY and ACSS2 control acetyl-CoA origin, shaping histone acetylation patterns and directing T cells toward progenitor-like or terminal exhaustion (16). AMPK phosphorylates the FOXO1-TCF1 axis, influencing the balance between metabolic state and exhaustion fate (49).

Collectively, metabolite-sensitive PTMs link nutrient availability to transcriptional and functional changes during exhaustion.

### Epigenetic regulators

Once established, CD8^+^ T cell exhaustion exhibits high stability and remains difficult to fully reverse even after antigen clearance or inhibition of signal release. This stability is essentially determined and maintained by the epigenetic landscape (50). Epigenetic regulation mainly shapes stable gene expression patterns through three mechanisms. These mechanisms are DNA methylation, chromatin remodeling, and histone modification (as detailed in the ‘Epigenetic Memory Locking’ section). Through these mechanisms, epigenetic regulation locks the exhaustion program for a long time.

PTMs regulate the activity, localization, and stability of epigenetic enzymes, chromatin remodelers, and methylation- and histone-related proteins via phosphorylation, ubiquitination, and glycosylation. This enables rapid writing of extracellular signals and metabolic states into chromatin. PTM interactions jointly regulate epigenetic factors, laying the foundation for converting transient signals into stable epigenetic memory.

## Individual PTM types in exhaustion

We have shown how PTMs regulate four core functional modules. Five PTMs, namely phosphorylation, ubiquitination, acetylation, glycosylation, and lactylation, are selected as “key” based on three criteria: 1) their prevalence and depth of characterization in the CD8^+^ T cell exhaustion literature; 2) their mechanistic relevance to the signal, metabolism, and epigenetic axis, as each participates in at least two of the four core functional modules; and 3) their therapeutic tractability, as pharmacological modulators for these pathways are already in clinical development or represent promising drug targets. Next, we discuss the distinct roles of individual PTM types, starting with phosphorylation, the earliest and fastest responding modification in the exhaustion program.

### Phosphorylation: core switch of receptor signal hub and exhaustion initiation

Phosphorylation is the earliest activated modification in the PTM-driven model. It converts sustained TCR signals into intracellular cascades and serves as the initiating switch of the exhaustion program, laying the foundation for metabolic reprogramming and epigenetic regulation. Through kinase-phosphatase antagonism, phosphorylation achieves rapid signal activation and inactivation, integrating TCR, cytokine, and metabolic signals into a coordinated exhaustion program (51). As the fastest and most broadly distributed PTM, it operates across membrane signaling, transcription, metabolism, and epigenetic regulation, thus facilitating subsequent synergistic modifications.

At the level of membrane receptors and proximal signals, persistent TCR signals are the source of exhaustion initiation, which can continuously activate key kinases such as LCK and ZAP-70, and promote the upregulation of IRs such as PD-1, CTLA-4, and TIM-3 (52). After PD-1 binds to its ligands, the ITIM (Y223) and ITSM (Y248) motifs in its intracellular domain become phosphorylated. This recruits the phosphatase SHP-2. SHP-2 then dephosphorylates ZAP-70 and blocks downstream TCR signaling (53). Concurrently, ERK-mediated phosphorylation of PD-1 at Thr234 recruits the deubiquitinase USP5. It removes K48-linked polyubiquitin chains and stabilizes the PD-1 protein, thereby prolonging its membrane retention time and enhancing inhibitory signal output (31).

At the transcriptional regulation level, phosphorylation controls exhaustion gene expression by regulating transcription factor nucleocytoplasmic shuttling, DNA binding, and activity. Calcium signaling triggers NFAT dephosphorylation and nuclear entry, followed by re-phosphorylation by GSK-3β and CK1, which sustains PDCD1 and TOX expression (54). At the same time, cytokine signals are integrated and transmitted through the phosphorylation state of STAT family proteins. Specifically, the phosphorylation levels of STAT1/3 increase and the phosphorylation level of STAT5 decreases, which together drive the transcriptome to tilt toward exhaustion and further stabilize the exhaustion program (55–58).

At the level of metabolism and nutrient sensing, phosphorylation directly dominates the direction of metabolic reprogramming. The PI3K/Akt/mTOR signaling axis functions normally in effector T cells. In Tex, this axis undergoes abnormal phosphorylation in Tex, leading to the failure to activate key glycolytic enzymes and insufficient energy supply (59). When decreased ATP levels trigger energy stress, AMPK is phosphorylated and activated at Thr172, transiently promoting PGC-1α expression to maintain mitochondrial biogenesis. However, under persistent stress, this pathway is eventually inactivated. This leads to mitochondrial dysfunction, ROS accumulation, and metabolic collapse. These changes further exacerbate T cell exhaustion (44, 45, 60).

At the epigenetic regulation level, phosphorylation paves the way for the final epigenetic solidification of the exhaustion program. It regulates the activity, stability, and nuclear localization of epigenetic modification enzymes. Phosphorylation modifies key epigenetic enzymes such as EZH2, HDACs, and DNMTs. This promotes H3K27me3 deposition, enhances histone deacetylation, and stabilizes DNA methylation. These changes gradually silence effector genes and promote the exhaustion program toward a stable and irreversible direction (61–64).

Collectively, phosphorylation holds a central position in the PTM network: it initiates earliest, responds fastest, and spans membrane signaling, transcription, metabolism, and epigenetics. It also provides the upstream foundation for ubiquitination, glycosylation, and acetylation, serving as the initiation hub of the exhaustion regulatory network.

### Ubiquitination and UBLs: core hub of protein homeostasis and signal stability

Following phosphorylation, ubiquitination and UBLs maintain protein homeostasis, stabilize inhibitory signals, and sustain the exhaustion program. Unlike phosphorylation, the ubiquitination system converts transient signals into persistent protein-level changes by regulating protein stability, degradation, and complex assembly. It forms a synergistic network with phosphorylation, glycosylation, and acetylation. Ubiquitination is catalyzed by E1-E2-E3 enzymes and reversed by DUBs, determining the fate of key proteins through degradation or signal modulation.

### Ubiquitination modification

At the level of membrane receptors and proximal signals, ubiquitination directly determines the membrane abundance and signal intensity of IRs, serving as an important continuation and enhancement of phosphorylation signals. E3 ubiquitin ligases FBXO38 (65) and KLHL22 (66) promote PD-1 polyubiquitination and degradation, limiting its accumulation under normal conditions. In the TME, this regulation is inhibited, leading to PD-1 stabilization and sustained inhibitory signaling (67). It should be noted, however, that the role of FBXO38 in PD-1 regulation has recently been challenged. A study by Dibus et al. reported that FBXO38 does not interact with PD-1 and that FBXO38 deficiency does not affect PD-1 surface levels in T cells under steady-state, ex vivo activation, or viral infection conditions (68). These conflicting findings suggest that the ubiquitination landscape of PD-1 may be more complex than initially appreciated, and that additional factors or context-dependent mechanisms may be involved. Unlike the degradation-type regulation of PD-1, LAG-3 can directly undergo conformational activation through K63-linked non-degradative ubiquitination. This initiates its inhibitory functions without the proteasome pathway (69). More importantly, Cbl-b forms a complex with STS1, simultaneously ubiquitinating and dephosphorylating CD3ζ and ZAP-70, thereby blocking TCR signaling through both protein stability and activity (70).

At the transcriptional regulation level, ubiquitination shapes the exhaustion-specific program by controlling transcription factor stability and balance. KLHL6, an E3 ligase, assembles with Cullin3 and Rbx1 to catalyze K48-linked polyubiquitination of TOX, targeting it for degradation. Persistent TCR signaling suppresses KLHL6 via the PI3K-AKT-FOXO1 axis, reducing TOX ubiquitination and promoting its accumulation, which accelerates Tpex-to-Tex differentiation. KLHL6 also targets PGAM5 for degradation; its downregulation causes PGAM5 accumulation and Drp1-mediated mitochondrial fragmentation. Thus, TOX accumulation drives exhaustion while PGAM5 accumulation causes mitochondrial dysfunction, synergistically promoting Tex exhaustion (39, 71). Additionally, ubiquitination degrades AP-1 members like c-Jun, impairing effector function and promoting NFAT-driven exhaustion (72). USP8 stabilizes TβRII, enhancing SMAD signaling and forming an immunosuppressive loop (73).

At the level of metabolism and mitochondrial homeostasis, ubiquitination and UBLs regulate metabolic reprogramming and mitochondrial quality, linking signal networks to metabolic collapse. The VHL E3 complex controls HIF-1α ubiquitination and degradation, affecting glycolysis. Mitochondrial oxidative stress inhibits HIF-1α degradation, promoting Tpex-to-Tex transition (74, 75). Meanwhile, the deubiquitinase USP30 inhibits Parkin-mediated mitophagy. This leads to the massive accumulation of damaged mitochondria and excessive release of ROS, which further exacerbates metabolic dysfunction (76). This series of regulations enables the ubiquitination system to convert signal abnormalities into persistent metabolic defects.

At the level of epigenetic paving, ubiquitination directly participates in the establishment of histone modifications, laying the foundation for subsequent epigenetic locking. Monoubiquitination of histone H2AK119 (H2AK119Ub) mediated by the PRC1 complex can construct an inhibitory chromatin structure and silence effector-related genes (77). Deubiquitination mediated by ASXL1 can remove this mark, maintaining the stemness and plasticity of Tpex cells (78). The dynamic balance between the two determines whether the exhaustion program is in a plastic state or gradually becomes solidified.

### UBLs

The ubiquitination system has important extended forms. SUMOylation, NEDDylation and UFMylation belong to UBLs. These modifications function beyond classic ubiquitin degradation, complementing the PTM network in protein regulation, metabolic homeostasis, and stress response.

SUMOylation negatively regulates immune function by modifying transcription factors and metabolic molecules. In exhausted CD8^+^ T cells, SUMOylation modulates T cell differentiation via Cbx4, a Polycomb-group protein whose SUMO-interacting motifs repress memory phenotype and sustain T-bet/Eomes balance (79) At the same time, SUMOylation also reduces glycolysis and mitochondrial respiration rates, putting cells into a low-metabolic state, and becomes one of the important reasons for mediating PD-1 inhibitor resistance (80). In models such as lymphoma and pancreatic cancer, the use of SUMOylation inhibitors such as TAK-981 can reprogram the metabolism of exhausted TILs, restore cytotoxicity, and synergize with immune ICB to improve therapeutic efficacy (81, 82).

NEDDylation occupies a core position in metabolic regulation and protein homeostasis. Its main function is to activate the Cullin-RING ubiquitin ligase (CRLs) family, indirectly regulating the ubiquitination and degradation of a large number of downstream proteins (83, 84). More importantly, NEDDylation can directly modify core glycolytic enzymes such as LDHA, ENO1, and HK1, directly determining the energy production efficiency and metabolic adaptability of CD8^+^ T cells. It is crucial for cell survival, expansion, and anti-tumor ability in the tumor microenvironment (85).

UFMylation, a UBL localized to the ER, regulates ER-phagy and protein homeostasis (86, 87). The UFMylation pathway maintains ribosome function and endoplasmic reticulum stress tolerance. This inhibits the transformation of CD8^+^ T cells into an exhausted-like phenotype by reducing IR upregulation and epigenetic remodeling. It serves as an important defense mechanism for cells to resist microenvironmental stress and delay the exhaustion process (86, 87). These three UBLs cooperate with ubiquitination to refine the exhaustion network via transcriptional inhibition, metabolic reprogramming, and ER homeostasis.

In conclusion, ubiquitination and UBLs play the roles of “signal stabilizers” and “program maintainers” in the entire PTM network. They take over the transient signals initiated by upstream phosphorylation, convert short-term signals into persistent functional changes by regulating protein stability and metabolic status. This provides an necessary intermediate state for downstream acetylation, lactylation, and epigenetic locking. They are the key hub for exhaustion to move from “initiation” to “maintenance” and “solidification”.

### Glycosylation: membrane signal sensor and metabolic-transcriptional coupler

After phosphorylation initiates exhaustion signals and ubiquitination stabilizes protein homeostasis, glycosylation assumes its role as a key metabolic sensor. As a PTM highly dependent on the nutritional state of the microenvironment, it further assumes the key roles of “extracellular signal sensor” and “metabolic-transcriptional bridge” (88). It comprises two major types: N-glycosylation of membrane proteins and O-GlcNAc glycosylation of intracellular proteins, which regulate receptor sensing and metabolic-transcriptional coupling, respectively. Glycosylation synergizes with phosphorylation and ubiquitination to translate glucose metabolism into CD8^+^ T cell fate decisions (89, 90).

At the level of membrane receptor and proximal signal regulation, N-glycosylation directly determines the conformation, ligand affinity, membrane localization, and signal transduction efficiency of IRs. It serves as an important regulatory link in the initiation and maintenance of exhaustion. FUT8-catalyzed core fucosylation of PD-1 enhances PD-L1 binding, stabilizes inhibitory signaling, and reduces antibody efficacy (91). Galectin-9 binds specifically to glycosylated TIM-3. This interaction requires proper N-glycosylation of the TIM-3 IgV domain. Upon binding, galectin-9 triggers TIM-3 mediated inhibitory signaling, which increases PD-1, TIM-3, and LAG-3 expression on CD8^+^ T cells while reducing IFN-γ production. This signaling axis operates independently of PD-1 and persists even during anti-PD-1 treatment, thereby contributing to immunotherapy resistance. Thus, TIM-3 glycosylation is essential for galectin-9 recognition and subsequent T cell exhaustion (92). Meanwhile, MGAT5-mediated N-glycan branching forms a membrane “lattice” that restricts TCR movement, raising the activation threshold and promoting exhaustion (93). More importantly, glycosylation also senses metabolic stress: nutrient scarcity reduces CD36 N-glycosylation, activating AMPK and destabilizing USP7, which promotes RBPJ degradation via a glycosylation-phosphorylation-ubiquitination cascade, inhibiting effector programs and accelerating exhaustion (94).

At the level of intracellular metabolic-transcriptional coupling, O-GlcNAc glycosylation dynamically connects glucose metabolism to transcription and epigenetic remodeling in the nucleus and cytoplasm, serving as a metabolic signal converter. In CD8^+^ T cells, O-GlcNAc modifies numerous signaling proteins, transcription factors, and epigenetic regulators involved in fate regulation (95). On the one hand, the O-GlcNAc transferase OGT can directly interact with DNA demethylases such as TET2 and TET3. Through glycosylation modification, OGT regulates their enzyme activity and chromatin localization. This affects the expression of memory-related genes and stemness maintenance (96, 97); on the other hand, mannose supplementation can enhance OGT-mediated β-catenin glycosylation, stabilize protein levels, and continuously maintain TCF-1 expression. Consequently, it helps retain the stemness and plasticity of progenitor-like Tex and weaken the terminal exhaustion process (98).

Overall, glycosylation plays dual roles of “membrane interface signal sensor” and “metabolic-transcriptional coupler” in the PTM network. It integrates microenvironmental signals at the membrane, couples nutrient metabolism to transcriptional and epigenetic outputs, and interacts with phosphorylation and ubiquitination to provide metabolic input for acetylation and lactylation-driven epigenetic locking, serving as a key node for exhaustion transition from “signal response” to “metabolic adaptation”.

### Acetylation: key bridge from metabolic flow to epigenetic memory

Acetylation directly uses acetyl-CoA as a donor, positioning it as the critical link between metabolism and epigenetics in the PTM-driven model. It “writes” transient metabolic fluctuations into stable chromatin marks and is dynamically regulated by HATs and HDACs (99). It acts on histones to reshape chromatin structure. And it also directly modify transcription factors and metabolic enzymes, establishing an irreversible reinforcement loop between metabolic signals, transcriptional programs, and epigenetic fate. It is the key hub that drives exhaustion from the “adaptation stage” to the “locking stage”.

At the level of metabolism-driven epigenetic remodeling, acetyl-CoA source and abundance determine acetylation site specificity and functional direction. In terminally Tex, ACLY produces acetyl-CoA from citrate, which KAT2A uses to deposit H3K27ac at exhaustion enhancers, reinforcing the program (16). In contrast, in progenitor-like exhausted or memory T cells, ACSS2 generates acetyl-CoA from acetate, favoring p300 and maintaining effector/stemness gene expression and plasticity (100, 101). This difference in acetylation distribution determined by metabolic sources selectively consolidates the exhaustion program and silences the effector program at the chromatin level. It is a key step in converting metabolic signals into epigenetic memory.

At the level of transcription factor regulation, acetylation directly determines the activity and functional stability of core transcription factors, further consolidating the exhaustion transcriptional network. TOX recruits the HBO1 acetylation complex. This promotes histone acetylation and continuously activates downstream exhaustion-related genes, forming a positive amplification loop (102). Meanwhile, SIRT1 maintains FoxO1 active to support stemness and memory; its decline under metabolic stress leads to FoxO1 inactivation and stemness loss, exacerbating exhaustion (103). Acetylation acts as a precise switch for transcription factor function. Consequently, it propels T cells from a stem-like progenitor state toward terminal exhaustion.

At the level of metabolic enzyme function regulation, acetylation inhibits core metabolic enzymes including GAPDH (104) and LDH (105), forming a negative feedback loop that exacerbates Tex energy deficiency. In Tex, accumulated acetylation reduces glycolytic efficiency and impairs mitochondrial respiration, trapping cells in an energy-depleted state. This metabolic insufficiency alters acetyl-CoA supply, further reinforcing epigenetic exhaustion and creating a vicious cycle: metabolic inhibition drives abnormal acetylation, which in turn promotes epigenetic locking.

Overall, acetylation plays dual roles of “metabolic signal integrator” and “epigenetic memory initiator” in the PTM network. It takes over the nutritional signals transmitted by upstream glycosylation, converts fluctuations in metabolic intermediates into site-specific histone and protein modifications. Through these modifications, it simultaneously strengthens exhaustion transcriptional programs such as TOX, inhibits metabolic enzyme functions. It thus lays a decisive foundation for terminal epigenetic locking mediated by downstream lactylation and methylation.

### Lactylation: direct epigenetic encoding of metabolic stress and exhaustion accelerator

Building upon acetylation, lactylation serves as the terminal metabolic stress sensor in the PTM-driven model. It directly “writes” lactate accumulation and acidosis into histones and functional proteins, serving as the most direct bridge between metabolic stress and epigenetic exhaustion. Lactylation modifies both histone and non-histone lysine residues, altering chromatin structure, transcription factor binding, and protein conformation to bidirectionally regulate CD8^+^ T cell effector function and metabolism, driving exhaustion into the terminal locking stage (106–108).

After entering CD8^+^ T cells through the monocarboxylate transporter MCT11, it directly increases histone lactylation levels (109). Elevated intracellular lactate serves as a direct donor for histone lactylation, which is catalyzed by p300 and removed by HDAC1-3. Specifically, H3K9la and H3K18la enrich at effector gene promoters (GZMB, PRF1, IFNG), reducing chromatin accessibility and suppressing transcription, thus promoting terminal exhaustion. The p300/HDAC1–3 balance determines silencing intensity (110). Meanwhile, lactylation also enhances METTL3 RNA binding, promoting m^6^A modification of JAK1 mRNA and sustaining STAT3 activation to reinforce immunosuppression and form a metabolism-driven positive feedback loop (111). Additionally, lactylation disrupts mitochondrial fission-fusion via Fis1 (112), causing fragmentation and metabolic dysfunction that impairs T cell recovery (113).

At the regulatory level of maintaining stemness and delaying exhaustion, the role of lactylation shows obvious cell state dependence, and instead exerts a protective effect in naive T cells and memory T cells. In resting naive CD8^+^ T cells, H3K9la mainly enriches in the regions of genes that maintain quiescence and stemness, such as Bach2, Foxo1, and Klf2. In memory T cells, lactylation marks tend to target stemness-maintaining genes such as Lef1, Ccr7, and Il7r. This suggests that moderate lactylation helps retain the progenitor-like self-renewal potential and balance the fate choice between self-renewal and terminal differentiation (110). Studies also found that inhibiting HDAC1~3 can increase the overall lactylation level and enhance the tumor-killing ability of T cells, further indicating that the function of lactylation has high site and context specificity (114).

Overall, lactylation plays dual roles of “metabolic stress sensor” and “terminal exhaustion accelerator” in the entire PTM regulatory network. It converts microenvironmental lactic acid signals into epigenetic marks in the most direct way, takes over the metabolic-epigenetic pathway of upstream acetylation, and simultaneously cross-talks with phosphorylation and ubiquitination signals. This process jointly promotes the exhaustion program toward irreversible epigenetic locking, and serving as a key driver for the stable maintenance of the terminally exhausted phenotype.

### Unconventional PTMs: expansion and fine regulation of the regulatory network

Beyond the five core modifications that form the backbone of our PTM-driven model, unconventional PTMs such as methylation, succinylation, and palmitoylation provide fine-tuning at key regulatory nodes. The five key PTMs were selected for their broad action spectra across the four functional modules: phosphorylation and ubiquitination span signaling, transcription, metabolism, and epigenetics; glycosylation bridges membrane sensing and metabolic coupling; acetylation and lactylation integrate metabolism with epigenetic outputs. Methylation, by contrast, acts predominantly within the epigenetic module (115). We therefore classify it as “unconventional” not because it is uncommon, but because its role in this framework is more narrowly focused on terminal chromatin stabilization. Thus, methylation functions as a “terminal locker” rather than a “hub” modification. Collectively, these unconventional modifications complement the mainstream PTM network by extending regulation across the signal-metabolism-epigenetic axis.

### Methylation: chromatin remodeling and epigenetic locking

Methylation is a stable and persistent covalent modification that mainly occurs on lysine and arginine residues. It participates in the long-term maintenance and epigenetic solidification of the exhaustion program by regulating chromatin structure and gene accessibility. During the exhaustion process of CD8^+^ T cells, histone methylation plays the role of a “stable silencer”. The histone methyltransferase SUV39H1 can catalyze the deposition of the inhibitory mark H3K9me3 in the regions of memory-related genes such as Tcf7, directly suppressing the stemness program and promoting cells toward terminal exhaustion (116). Meanwhile, as a core component of the PRC2 complex, EZH2 silences effector genes and memory genes by catalyzing the inhibitory mark H3K27me3, making it a key executor of epigenetic locking. At the clinical translation level, the EZH2 inhibitor Tazemetostat blocks inhibitory methylation marks. This partially reverses the exhausted phenotype of CD8^+^ T cells, restores stem cell characteristics, and enhances the efficacy of adoptive cell therapy (117). Methylation thus serves as more than a transient signal regulator. It represents an epigenetic mechanism that stably solidifies the exhaustion program.

### Succinylation: metabolite-driven enzyme activity regulation

Succinylation is a modification highly dependent on cellular metabolic status. It uses succinyl-CoA as a direct donor and changes enzyme activity, protein stability, and signal transduction ability by modifying lysine residues, directly converting the level of metabolic intermediates into functional output. In the tumor microenvironment, high concentrations of fumarate can directly perform inhibitory succinylation modification on ZAP-70, preventing its normal activation. This blocks downstream TCR signaling and rapidly impairs CD8^+^ T cell recognition and activation (118). In addition, CPT1A can act as a specific succinyltransferase of PD-L1, preventing PD-L1 degradation through succinylation modification, increasing the level of PD-L1 on the membrane surface, and strengthening tumor immune escape (119). These findings indicate that succinylation not only regulates metabolic enzyme activity, but also directly participates in the cross-regulation of immune checkpoint pathways and TCR signals. Thus, it represents an important pathway for metabolic stress-mediated immune suppression.

### Palmitoylation: membrane localization, stability, and signal integration

Palmitoylation is a reversible lipid modification that regulates protein membrane localization, transport, aggregation, and stability, orchestrating membrane signaling, mitochondrial metabolism, and cross-compartment communication. DHHC9-mediated TIM-3 palmitoylation enhances its stability and membrane localization, sustaining inhibitory signals and promoting exhaustion (120). Tumor-derived palmitic acid enhances STAT3 palmitoylation and persistent activation, driving Tpex-to-Tex transition (121). DHHC5-mediated SDHB palmitoylation alters mitochondrial metabolism, triggering H3K27ac modification and PD-1 upregulation, forming a cross-compartment axis from mitochondria to epigenetics to immune checkpoint expression (122). Therefore, palmitoylation is a key cross-boundary modification connecting membrane signals, mitochondrial metabolism, and epigenetics, significantly expanding the regulatory dimension of the PTM network.

Although unconventional PTMs each have their own focuses, they all deeply synergize with the mainstream modification network. Methylation is responsible for the final epigenetic locking, succinylation transmits metabolic stress signals, and palmitoylation regulates membrane localization and cross-compartment crosstalk. Together, they improve the PTM regulatory network of CD8^+^ T cell exhaustion, making the entire regulatory system more stable, precise, and irreversible, and providing the final key support for the final epigenetic memory locking.

## Integrated PTM regulatory network

The regulatory roles of individual PTM types described above are not isolated. During CD8^+^ T cell exhaustion, distinct PTMs form a complex regulatory network through synergistic or antagonistic interactions, jointly driving the initiation and locking of the exhaustion program. Having detailed the individual contributions of each PTM type to the four functional modules, we now integrate them into a unified regulatory network. The PTM-driven signal–metabolism–epigenetic locking model operates not through isolated modifications but through the spatiotemporal synergy, cascading, feedback, and cross-talk of different PTM types. These interactions collectively determine whether CD8^+^ T cells enter a reversible progenitor-like exhaustion state or proceed toward irreversible terminal exhaustion.

### Three core modes of PTM interactions

#### Phosphorylation-ubiquitination cascade regulation

Phosphorylation and ubiquitination form a classic cascade controlling protein stability and signal termination. Kinase-mediated phosphorylation creates E3 ligase recognition sites for substrate degradation, while DUBs maintain phosphorylation by stabilizing kinases, enabling bidirectional regulation. In CD8^+^ T cells, this cascade calibrates IRs and proximal signals: ERK-mediated PD-1 phosphorylation recruits USP5 for deubiquitination, enhancing PD-1 stability and prolonging inhibition; Cbl-b synergizes with phosphorylation to ubiquitinate and inhibit CD3ζ and ZAP-70, blocking TCR signals (70). Metabolic stress inhibits CD36 glycosylation, which activates AMPK. This further reduces ultimately strengthening the exhausted phenotype through the phosphorylation-ubiquitination-transcription axis (94).

#### Metabolite-driven epigenetic modification integration

Multiple PTMs use metabolic intermediates as direct donors, linking metabolism to epigenetics. Acetyl-CoA, lactate, succinyl-CoA, and UDP-GlcNAc serve as donors for acetylation, lactylation, succinylation, and O-GlcNAcylation, respectively, converting nutrient abundance into modification intensity. Lactate-derived H3K9la and H3K18la enrich at effector gene promoters (GZMB, PRF1, IFNG), reducing chromatin accessibility and suppressing transcription. These marks are catalyzed by p300 and removed by HDAC1-3, directly linking lactate metabolism to epigenetic silencing in exhausted CD8^+^ T cells (110, 114). Such metabolite-driven PTM interactions can quickly stabilize the exhaustion program without relying on *de novo* transcription synthesis, serving as a key pathway connecting metabolic stress and epigenetic locking.

#### DUBs-kinase positive feedback loop

Kinases and DUBs can form a self-reinforcing positive feedback loop that persistently sustains inhibitory signals. Kinases regulate the activity and localization of DUBs through phosphorylation. In turn, DUBs stabilize kinases or receptors via deubiquitination, preventing their degradation and thereby maintaining the inhibitory pathway in a sustained “on” state. For example, USP8 stabilizes the TGF-β receptor TβRII through deubiquitination, enhancing SMAD signaling and further exacerbating exhaustion (73). USP30, a mitochondrial deubiquitinase that inhibits mitophagy, is upregulated in exhausted CD8^+^ T cells and promotes the accumulation of dysfunctional mitochondria and elevated ROS levels, which in turn sustains phosphorylation signaling; however, its complete loss may impair sustained cytotoxic function by limiting granzyme B synthesis, suggesting a complex, context-dependent role in exhaustion (76, 123). Such feedback loops make exhaustion difficult to terminate once initiated, representing a key reason for the poor reversibility of Tex cell dysfunction.

### Cross-compartmental crosstalk of PTMs

The PTM network operates across multiple cellular compartments, including the plasma membrane, cytoplasm, nucleus, and mitochondria, to achieve cell-wide signal integration, collectively driving the progression of exhaustion.

At the plasma membrane, glycosylation and palmitoylation regulate receptor conformation, localization, and ligand affinity, thereby determining the intensity of incoming signals. Within the cytoplasm, phosphorylation and ubiquitination transmit and amplify signals, modulating the activity of kinases, transcription factors, and metabolic enzymes. In the nucleus, acetylation, lactylation, ubiquitination, and methylation collectively remodel chromatin accessibility. Within mitochondria, NEDDylation, palmitoylation, and phosphorylation of metabolic enzymes alter energy supply, which in turn reciprocally regulates nuclear epigenetic states. A representative cross-compartmental axis is the DHHC5-mediated palmitoylation of succinate dehydrogenase B (SDHB). This modification leads to aberrant mitochondrial metabolism, which subsequently increases H3K27ac levels and upregulates PD-1 expression, directly driving exhaustion from the metabolic perspective (122). This axis exemplifies the high degree of coordination within the PTM network across different cellular compartments.

### Context-dependent and divergent PTM functions

While our PTM-driven model provides a cohesive framework, several lines of evidence indicate that PTM functions are highly context-dependent and occasionally divergent.

First, ubiquitination can produce opposing outcomes. FBXO38-mediated PD-1 ubiquitination promotes its degradation, yet a recent study reported conflicting findings on PD-1 ubiquitination dynamics (68), suggesting the regulatory landscape is more complex than initially appreciated. Second, lactylation exhibits context-dependent duality. It suppresses effector gene transcription in terminally exhausted T cells but supports stemness gene expression in naive and memory T cells. Glycosylation similarly shows opposing effects, as it can either enhance PD-1 inhibitory signaling or promote TCR activation depending on the glycoprotein and glycan structure involved. Third, the TOX-TCF-1 relationship is nuanced. While TOX drives terminal exhaustion and TCF-1 maintains stemness, these factors can co-exist in progenitor-like exhausted T cells, with their relative abundance determining differentiation trajectories. Fourth, the temporal staging of PTM dominance represents a conceptual framework. These transitions likely occur along a continuum with considerable overlap, and precise timing may vary across experimental systems.

Collectively, these observations underscore that the PTM network operates through a complex balance of opposing modifications rather than unidirectional regulation, highlighting the need for site-specific and context-dependent analyses in future studies.

### Temporal dynamics of the PTM network

**Figure 2:**
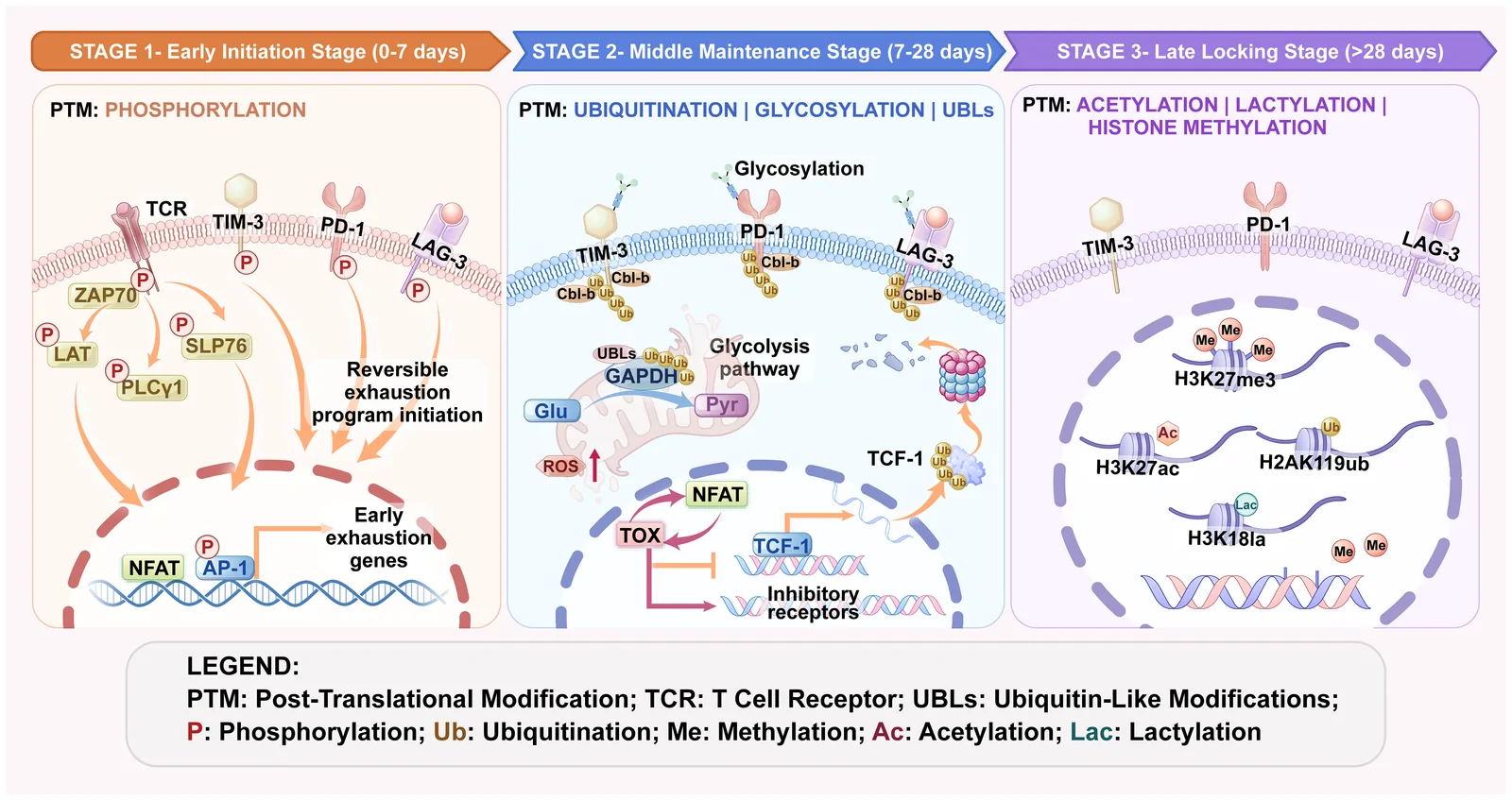
Temporal dynamics of the PTM network during CD8^+^ T cell exhaustion. The PTM-driven signal–metabolism–epigenetic locking model exhibits a three-stage temporal progression. In the early initiation stage (0–7 days post-chronic stimulation), phosphorylation (red) dominates to transmit persistent TCR signals, upregulate IRs, and initiate the exhaustion program (reversible). In the middle maintenance stage (7–28 days), ubiquitination, glycosylation, and UBLs (blue) stabilize IRs, remodel transcription factor balance, and reprogram cellular metabolism (partially reversible). In the late locking stage (>28 days), acetylation, lactylation, and histone methylation (purple) consolidate transient signals into durable epigenetic memory, silence effector genes, and render exhaustion irreversible. This dynamic transition converts functional inhibition into permanent cell fate determination.

During different stages of CD8^+^ T cell exhaustion, the dominant types of PTMs exhibit a characteristic pattern of transition, forming a complete temporal sequence that proceeds from initiation to maintenance and finally to locking (**Figure 2**). In the early initiation stage, phosphorylation dominates. It rapidly transmits sustained TCR signals, upregulates IRs, and initiates the exhaustion program. In the middle maintenance stage, ubiquitination, glycosylation, and UBLs (UBLs) take the lead. These modifications stabilize IRs, regulate the balance of transcription factors, and remodel metabolism. In the late locking stage, acetylation, lactylation, histone methylation, and H2AK119ub prevail. These modifications consolidate transient signals into durable epigenetic memory, rendering exhaustion irreversible. This dynamic transition demonstrates that the PTM network is not a static structure but is continuously remodeled as exhaustion progresses. Ultimately, this process achieves a complete transition from functional inhibition to epigenetic locking.

Through cascades, metabolic coupling, positive feedback loops, and cross-compartmental regulation, various PTMs form a highly integrated signaling network. This network connects the four major functional modules including membrane receptors, transcription, metabolism, and epigenetics into a unified exhaustion program. The temporal dynamics and synergistic interplay of the PTM network collectively determine whether CD8^+^ T cells enter a reversible progenitor-like exhaustion state or proceed toward irreversible terminal exhaustion.

## Epigenetic memory locking: terminal consolidation of the exhaustion program

**Figure 3:**
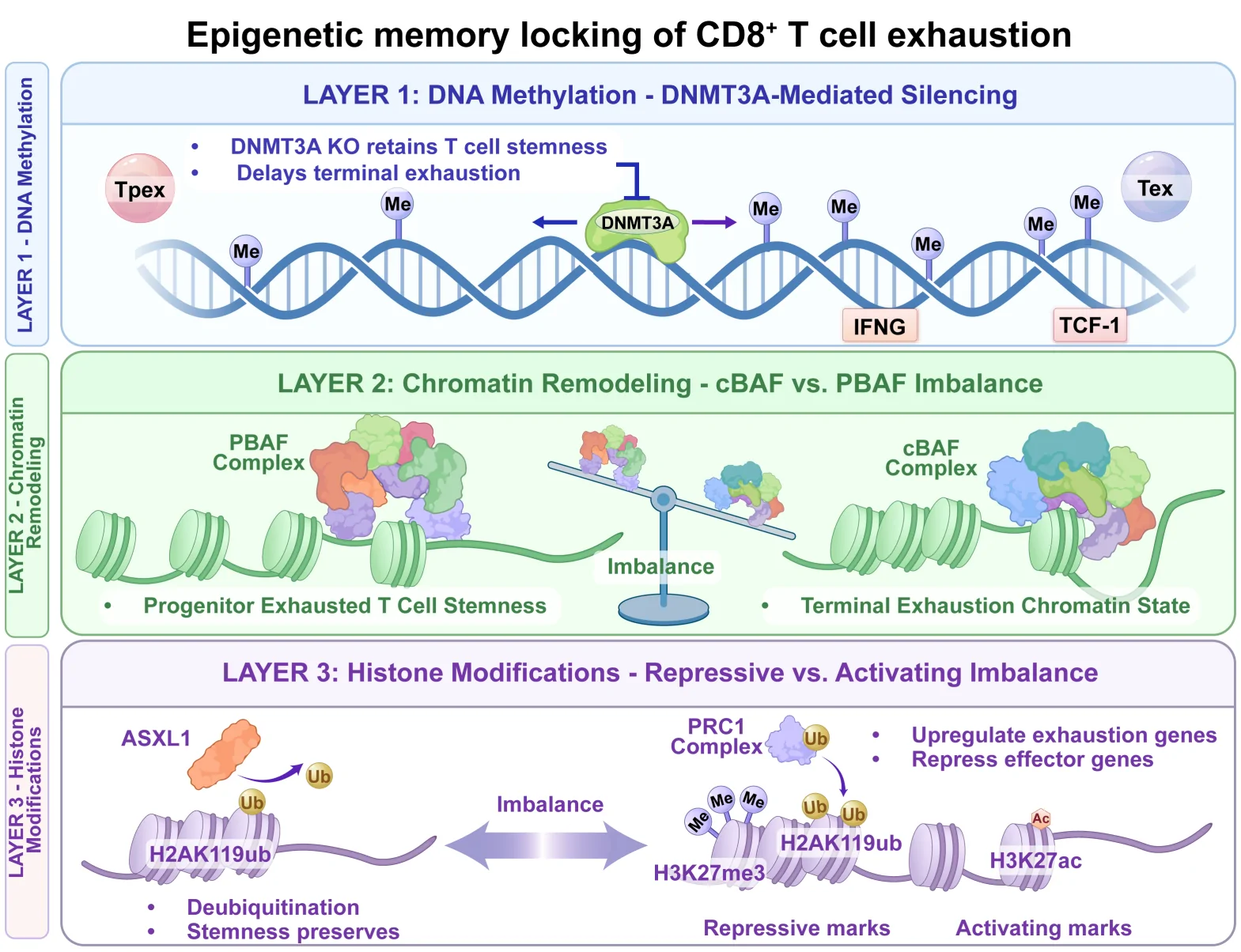
Epigenetic memory locking of CD8^+^ T cell exhaustion: three core layers and their PTM inputs. The three epigenetic layers herein represent chromatin regulatory mechanisms, not the three PTM-classified temporal exhaustion stages shown in **Figure 2**. The three temporal stages correspond to plastic Tpex, mixed intermediate subsets, and irreversible terminal Tex respectively. The schematic illustrates the terminal output of the PTM-driven model, converting short-term PTM signals into long-term cell fate decisions. Three core epigenetic mechanisms are color-coded: DNA methylation in blue, chromatin remodeling in green, and histone modifications in purple. Dashed arrows indicate the causal cascade: PTM inputs–epigenetic enzyme regulation–epigenetic modifications–gene silencing. First layer (DNA methylation, blue): DNMT3A silences effector and stemness genes via *de novo* methylation. PTM inputs (phosphorylation, ubiquitination) regulate DNMT3A activity and stability. Second layer (chromatin remodeling, green): The cBAF/PBAF balance determines exhaustion fate, with cBAF promoting terminal exhaustion and PBAF maintaining stemness. PTM inputs (phosphorylation, ubiquitination, glycosylation) modulate complex assembly and activity. Third layer (histone modifications, purple): Repressive marks (H3K27me3, H2AK119ub) silence effector genes; activating marks (H3K27ac, H3K9la, H3K18la) promote exhaustion gene expression. PTM inputs are metabolite-driven: acetylation and lactylation write metabolic signals into epigenetic memory; ubiquitination modifies histones and stabilizes PRC1; phosphorylation regulates HDACs and TET2.

As the terminal output of the PTM-driven model, epigenetic locking converts short-term PTM regulatory signals into long-term, stable cell fate decisions, serving as the core mechanism underlying the irreversibility of CD8^+^ T cell exhaustion. Epigenetic memory locking uses three core mechanisms to convert transient signals, metabolic shifts, and transcriptional changes into stable chromatin marks. This process progressively consolidates the exhaustion program. Thus, CD8^+^ T cells lose the ability to regain effector function (**Figure 3**). Notably, the three epigenetic regulatory layers in **Figure 3** are static chromatin-locking machineries, which are distinct from the three temporal exhaustion stages (**Figure 2**) divided by dominant PTMs. The three temporal stages bear distinct phenotypes: early-stage plastic Tpex with high TCF-1 and partial effector function; middle-stage mixed Tpex/intermediate exhausted cells with elevated TOX and inhibitory receptors; late-stage terminal Tex with silenced cytotoxic genes and lost plasticity.

The first layer is DNA methylation. DNMT3A-mediated *de novo* methylation silences effector and stemness genes, consolidating exhaustion, while DNMT3A deletion preserves stemness and delays terminal exhaustion (124, 125). This mechanism confers long-term stability to the exhaustion program at the DNA level. The second layer is chromatin remodeling. The cBAF/PBAF balance determines Tex fate: cBAF promotes terminal exhaustion, whereas PBAF maintains progenitor-like features (126). The third layer is histone modifications. Repressive marks (H3K27me3, H2AK119ub) and activating marks (H3K27ac) selectively regulate exhaustion versus effector genes. PRC1-mediated H2AK119ub establishes repressive chromatin, while ASXL1-mediated deubiquitination removes it to preserve Tpex stemness and plasticity (77, 78).

Various PTMs drive this locking process through specific pathways. Phosphorylation regulates the activity of epigenetic enzymes to initiate locking. Ubiquitination directly modifies histones to consolidate the repressive state. Acetylation and lactylation write metabolic signals into epigenetic memory. Atypical modifications such as glycosylation provide auxiliary support through cross-compartmental signaling. The temporal staging of PTM dominance (early phosphorylation, middle ubiquitination/glycosylation, late acetylation/lactylation/methylation) is a theoretical integration model; however, longitudinal multi-PTM tracking in human tumor-infiltrating CD8^+^ T cells remains insufficient for full validation, representing a current limitation. Ultimately, epigenetic locking transforms CD8^+^ T cell exhaustion from a reversible state of functional inhibition into a consolidation of cellular fate that is difficult to fully reverse. This transformation represents an important contributing factor of tumor immune evasion and resistance to immunotherapy, while also providing core targets for future epigenetic interventions aimed at reversing exhaustion.

## Therapeutic implications and perspectives: translating the PTM network into clinical practice

Building upon the PTM-driven signal–metabolism–epigenetic locking model established, we now delineate three key translational avenues that harness PTM regulatory networks to overcome CD8^+^ T cell exhaustion and enhance cancer immunotherapy.

### PTM-targeting agents combined with ICB: a dual-intervention strategy

PTM inhibitors can synergize with ICB through three complementary mechanisms: reversing T cell exhaustion, reshaping the tumor microenvironment, and enhancing antigen presentation. Among the most promising classes of PTM-targeting agents are SHP-2 inhibitors, SUMOylation inhibitors, and HDAC inhibitors.

SHP-2 inhibitors (e.g., TNO155) block the phosphatase recruited by phosphorylated PD-1 to terminate TCR signals (127, 128). Preclinical studies have demonstrated that SHP-2 inhibition restores CD8^+^ T cell effector function and reduces immunosuppressive myeloid cell infiltration (129). Several phase I/II trials are currently evaluating SHP-2/PD-1 combinations in solid tumors (130). SUMOylation inhibitors (TAK-981) reprogram exhausted CD8^+^ tumor-infiltrating lymphocyte metabolism by increasing glycolytic and mitochondrial capacity while upregulating MHC-I expression on tumor cells (131). In a phase I/II trial for relapsed/refractory non-Hodgkin lymphoma, TAK-981 combined with rituximab achieved a 27.6% objective response rate, with most adverse events being transient flu-like symptoms (132). In pancreatic cancer models, TAK-981 combined with anti-TIGIT significantly prolonged survival and generated protective immune memory (133). HDAC inhibitors enhance H3K9la and H3K18la levels to restore cytotoxic gene expression in Tex (110). A recent study further demonstrated that HDAC6 inhibitor ITF3756 downregulates PD-L1 expression on myeloid cells while enhancing their costimulatory capacity, thereby promoting T cell activation (134).

Nevertheless, several clinical challenges remain. First, combining HDAC inhibitors with ICB may increase the risk of immune-related adverse events due to additive toxicity. Second, robust biomarkers are urgently needed to identify patients most likely to benefit from these combination strategies. Third, the optimal sequencing of PTM inhibitors relative to ICB administration remains unclear.

### Engineering PTM-resistant CAR-T cells: from gene editing to smart receptors

The PTM model provides precise molecular targets for genetically engineering CAR-T cells that resist exhaustion and maintain long-term function. DNMT3A knockout preserves a progenitor-like, highly plastic T cell state and prevents terminal exhaustion, with whole-genome bisulfite sequencing confirming that DNMT3A deletion blocks the exhaustion-associated DNA methylation program in CAR-T cells during chronic stimulation (124). EZH2 inhibition (via tazemetostat) reduces regulatory T cells, promotes memory CAR-CD8 phenotypes, and alleviates exhaustion, thereby improving CAR-T efficacy in lymphoma models (135). USP30 knockout prevents mitophagy inhibition. Blocking USP30 restores mitochondrial quality control and reduces ROS-driven exhaustion (76).

Beyond single-gene editing, we propose a PTM-regulatory module-integrated CAR architecture. This next-generation design incorporates an inducible deubiquitinase domain (e.g., USP5) fused to the intracellular tail. Upon CAR engagement, the deubiquitinase is activated and removes inhibitory ubiquitin chains from key signaling molecules such as CD3ζ and ZAP-70, converting activation signals into sustained effector function. Epigenome editing offers advantages over traditional genome editing, including reversibility, dose-dependent regulation, and avoidance of permanent genomic alterations (136–138). However, potential risks remain, including long-term epigenetic changes that may predispose to abnormal proliferation and off-target editing-related adverse events (138).

### PTM-related biomarkers for immunotherapy response and resistance

PTM profiles on circulating or intratumoral CD8^+^ T cells can serve as predictive and pharmacodynamic biomarkers. For efficacy prediction, low TOX ubiquitination (reflecting high TOX protein stability) correlates with an exhausted, ICB-refractory state. This is because KLHL6-mediated TOX degradation normally prevents terminal exhaustion (39, 71). Peripheral T cell H3K9la and H3K18la levels associate with poor effector gene expression and reduced ICB response. These lactylation marks are catalyzed by p300 and removed by HDAC1-3, and their enrichment at effector gene promoters (GZMB, PRF1, IFNG) suppresses transcription (110, 114). PD-1 core fucosylation status influences ligand binding and antibody recognition. High core fucosylation (catalyzed by FUT8) predicts worse response to anti-PD-1 in several solid tumors by stabilizing PD-1 protein and enhancing its binding to PD-L1 (91).

For resistance, sustained STAT3 phosphorylation (p-STAT3 Y705) in CD8^+^ tumor-infiltrating lymphocytes correlates with terminal exhaustion and PD-1 blockade resistance, identifying patients who may benefit from JAK/STAT inhibitors (55–58). High EZH2 expression and elevated H3K27me3 marks at effector gene loci (GZMB, PRF1) are associated with epigenetic silencing of the cytotoxic machinery (116, 117). These findings provide actionable therapeutic targets for patients resistant to standard ICB therapy.

Emerging technologies are accelerating biomarker discovery. Single-cell PTM proteomics (e.g., CyTOF with PTM-specific antibodies) enables simultaneous mapping of multiple PTM markers on individual T cells (139). Spatial proteomics preserves tissue architecture and reveals how PTM profiles of T cells change depending on their proximity to tumor cells (140, 141). These technologies thus improve biomarker specificity and accuracy by capturing PTM heterogeneity at both single-cell and spatial resolution.

## Limitations and future directions

This review has several limitations: 1) Most current studies on PTM regulatory mechanisms are based on mouse models. The PTM regulatory patterns in human CD8^+^ T cells may exhibit species-specific differences, and the clinical applicability of the model requires further validation. 2) The crosstalk between different PTMs, such as the synergistic or antagonistic relationships between phosphorylation and ubiquitination, remains largely unknown. The precise mechanisms of network regulation have not yet been fully elucidated. 3) The exact temporal window and reversibility threshold of epigenetic locking remain unclear, making it difficult to accurately define the intervention window. 4) The impact of PTM modifications in other cells within the tumor microenvironment, such as macrophages and dendritic cells, on CD8^+^ T cell exhaustion has not been incorporated into the model.

Future directions: 1) Employ single-cell PTM proteomics, spatial transcriptomics, and other advanced technologies to map the PTM modification profiles of distinct CD8^+^ T cell subsets in human tumor tissues. 2) Develop PTM site-specific targeted agents, such as PD-1 Y223 phosphorylation inhibitors, to enhance therapeutic specificity. 3) Investigate the differential roles of PTM regulation in memory T cell formation versus exhaustion, providing a basis for long-lasting immunotherapies. 4) Explore the impact of gut microbial metabolites on PTM modifications in CD8^+^ T cells to refine the regulatory network of the model. 5) Conduct multi-center clinical studies to validate the clinical value of PTM-related biomarkers and the efficacy of combination therapeutic strategies.
